# Au Nanospirals Transferred onto PDMS Film Exhibiting Circular Dichroism at Visible Wavelengths

**DOI:** 10.3390/mi11070641

**Published:** 2020-06-29

**Authors:** Gaku Furusawa, Tetsuo Kan

**Affiliations:** Department of Mechanical and Intelligent Systems Engineering, Graduate School of Informatics and Engineering, The University of Electro-Communications, 1-5-1 Chofugaoka, Chofu-City, Tokyo 182-8585, Japan; gaku@ms.mi.uec.ac.jp

**Keywords:** circular dichroism, metamaterial, chirality, optical filter

## Abstract

We propose a thin, single-layered circular dichroic filter with Au nanospiral structures on a polydimethylsiloxane (PDMS) thin film that has strong circular dichroism at visible wavelengths. Au nanospiral structures with a diameter of 70 nm were fabricated by cryogenic glancing angle deposition on a substrate with a nanodot array template patterned with the block copolymer PS-PDMS. The Au nanospiral structures were transferred onto a transparent and flexible PDMS thin film to fabricate a thin, single-layered circular dichroic filter. The filter had a very large circular dichroism peak of −830 mdeg at 630 nm. The results show that the Au nanospiral structures transferred onto PDMS thin film exhibit large circular dichroism at visible wavelengths.

## 1. Introduction

Structures whose mirror images cannot be made to overlap by simple rotations or translations, such as the secondary structure of biomolecules, present either right-handed or left-handed structures and are called chiral structures [1,2]. Since chiral structures have different dielectric constants for right circularly polarized (RCP) and left circularly polarized (LCP) light, they exhibit circular dichroism, which is a difference in the transmittance between RCP and LCP light. Circular dichroism is usually very weak in the case of natural materials and mainly exhibits the largest value in the ultraviolet region [2]. In recent years, since they present optical characteristics whose amplitude is significantly larger than those of natural materials [2,3], artificial micro/nanostructures called metamaterials have been attracting much attention [1,2,3,4,5,6,7,8,9,10,11,12,13,14,15,16]. In particular, chiral plasmonic metamaterials achieve strong circular dichroism by either shaping the plasmonic material into a shape that does not have a mirror symmetry plane or arranging the plasmonic particles to from spiral-like shapes [2]. Nanosized metal chiral structures have circular dichroism in visible wavelength [6,8,9,13,14,15,17,18,19,20]. In particular, nanosized metal three-dimensional helical chiral structures exhibit very strong chirality under visible light [14,17,18,19,20]. Taking advantage of this remarkable performance, an optical filter that is very thin and has unprecedented performance could be achieved. By controlling the circular polarization, an optical isolator could be constructed, which is a very attractive technology because it has the potential to realize anti-reflection functions with a very thin structure. It can also be employed to construct single-layered circular dichroic filters for visible light, which has never been seen before. In a previous study, metal nanospiral structures were formed, released and dispersed into a liquid, and they exhibited large circular dichroism in the visible range [18]. Ni nanospiral structures formed on a transparent substrate were also reported to present an enhanced circular dichroism when subjected to a strong magnetic field [17]. However, although many examples of film-like configurations on hard substrates [7,21,22,23], or meta fluids which have chiral structure in a liquid [15,18,24], have been reported, there have been no reports of a response to visible light when transferred onto a flexible film.

**Figure 1:**
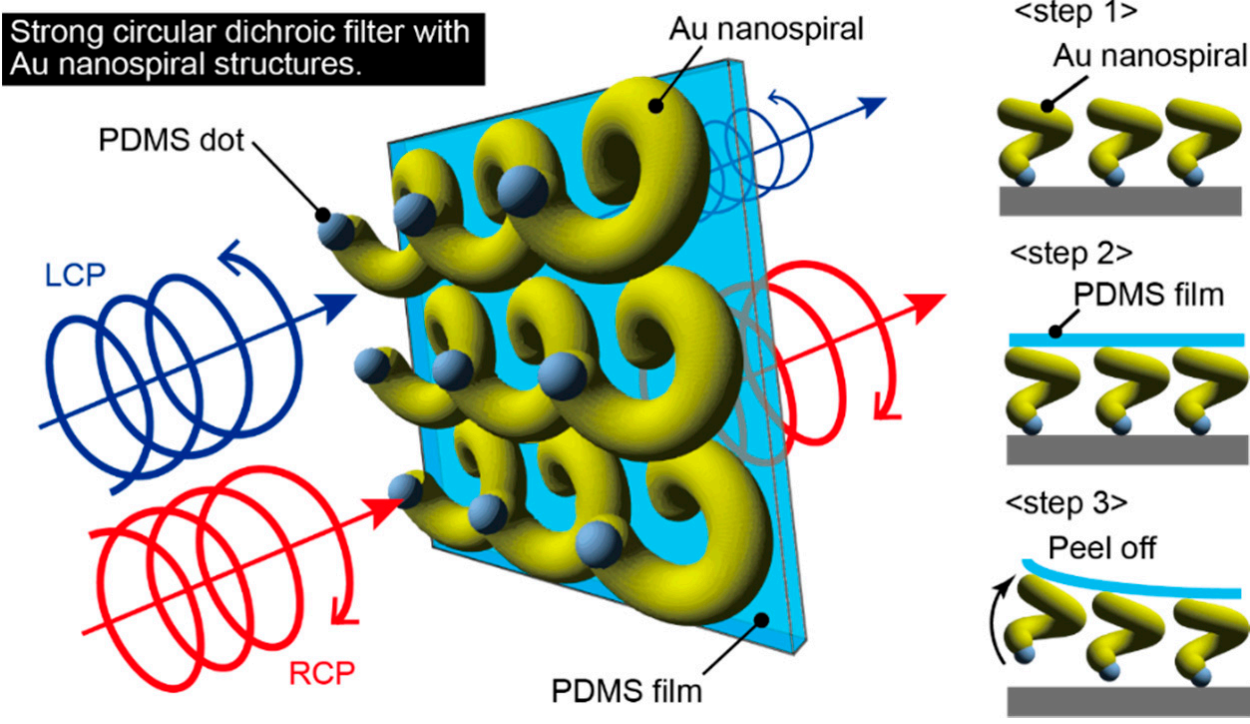
Proposed circular dichroic film at a visible wavelength with Au nanospiral structures transferred on a polydimethylsiloxane (PDMS) thin film.

In this paper, we describe a thin circular dichroic film in which an Au nanospiral structure is transferred onto a flexible and transparent polydimethylsiloxane (PDMS) thin film and examine its strong circular dichroism in the visible light region (Figure 1). We used this property to realize a circular dichroic film. In the device fabrication, nanodot templates were fabricated from block copolymers on a Si substrate. Three-dimensional Au nanospiral structures were fabricated by cryogenic glancing angle deposition on the nanodot template substrate. The nanospirals were selectively grown on each nanodot. A circular dichroic filter was fabricated by transferring the Au nanospiral structure onto a transparent flexible PDMS film to construct a film exhibiting circular dichroism. Measurement of the light transmitted through the fabricated circular dichroic film was performed, and strong circular dichroism was observed in the visible light range.

## 2. Fabrication

Au nanospiral structures were fabricated by cryogenic glancing angle deposition on a block copolymer nanodot array template formed on a Si substrate. A simple block copolymer, polystyrene–PDMS (PS-PDMS), was used to make a dot template to improve the accuracy of the three-dimensional helix formation. Au nanospiral structures were then transferred onto a PDMS film to construct a film exhibiting circular dichroism in the visible range.

### 2.1. Fabrication of Nanodot Templates Using Block Copolymers

**Figure 2:**
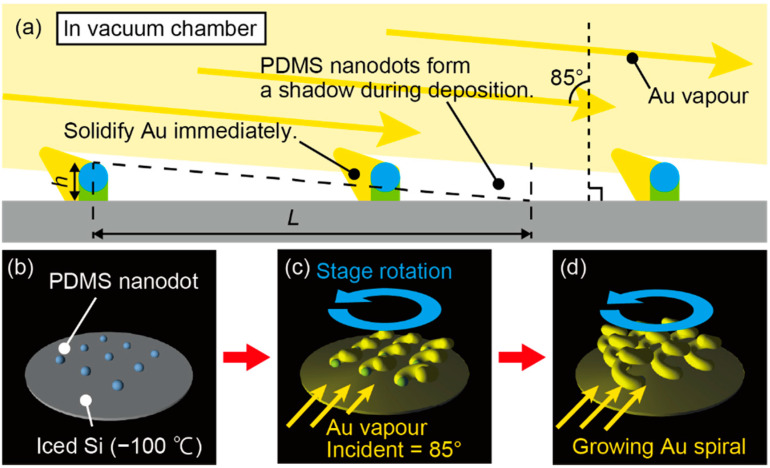
Overview of fabrication of Au nanospiral structures with cryogenic glancing angle deposition. (**a**) The PDMS nanodot templates are irradiated with Au vapor from 85°, the dots shadow each other, and Au is deposited at the tips of the dots. (**b**–**d**) Since the substrate is at an extremely low temperature, the Au solidifies instantaneously, and when the stage is rotated, a three-dimensional helical structure is formed.

Glancing angle deposition has been mainly used to fabricate nanoscale three-dimensional structures in dielectric materials [13,25,26,27,28], and its schematic for nanospiral formation is shown in Figure 2. When a noble metal, such as Au, is used as a deposition source in thermal evaporation, the metal vapor tends to form a continuous film after coming in contact with the substrate due to relocation of deposited metal. To inhibit this relocation and obtain noncontinuous separated metal structures, deposition was performed with cooling of the substrate [14,17,18]. The cooling of the substrate to cryogenic temperatures leads to immediate solidification of the metal vapor on the substrate and prevents the solidified metal vapor from forming a continuous film, providing island-like metal nanostructures. However, if cryogenic deposition is simply performed on a flat substrate, then the lateral spacing between the Au nano metal structures on the substrate becomes random, so the shape might be nonuniform and there is a risk that the structures will be connected to each other and become continuous. The effect of nanodot templates will be referred in later in our preliminary examination. It was found that without the nanodot template many metal structures tended to connect and could not form a clean spiral structure. We therefore employed Au nanodot templates on a Si substrate so that nanospiral structures can be formed with a moderately uniform lateral spacing. When the metal is deposited with an acute glancing angle, as shown in the upper part of Figure 2a, the nanodots shadow the metal vapor, and the metal vapor is thus selectively deposited on the nanodots. The shadow length *L* that the nanodot creates is expressed as

(1) $$L=\frac{h}{\tan\theta_{\mathrm{Au}}},$$

where $\theta_{\mathrm{Au}}$ is the angle at which the Au vapor is irradiated and *h* is the height of the nanodot. Since the dot template used in this paper is about *h* = 20 nm, considering the distance that each dot creates a shadow, the $\theta_{\mathrm{Au}}$ of about 80° < $\theta_{\mathrm{Au}}$ < 87° is expected to be suitable and was set to 85°. From the top view, cryogenic glancing angle deposition is performed with the nanodot template Si substrate being slowly rotated against the substrate surface around the vertical axis, as shown in Figure 2b. Then, as shown in Figure 2c, the metal vapor attaches to the apex of the nanodots and consolidates due to the cryogenic condition without relocation. Repeating this process leads to growth of the Au dots into nanospirals, as shown in Figure 2d. In the following, the detailed fabrication process is described.

**Figure 3:**
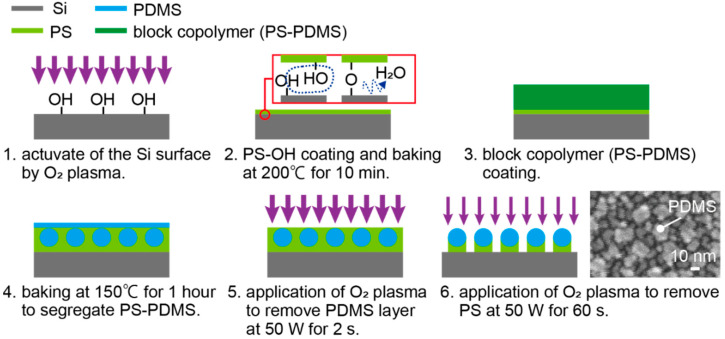
Patterning of nanosized dot templates on Si substrates using polystyrene–PDMS (PS-PDMS), a type of block copolymer.

The block copolymer PS-PDMS (P6194-SDMS Mn:47100-b-9000, Polymer Source Inc, Dorval, QC, Canada) and a hydroxy-terminated PS (P2996-SOH, Polymer Source Inc, Canada) were used for the fabrication of the nanodot template. The self-assembly of block copolymers is often used to fabricate nanosized large-area periodic nanostructures such as a nanodot array [29,30,31,32]. In particular, PS-PDMS is adopted in this work because it is advantageous for the fabrication of smaller dot patterns such as on the 10 nm scale [29,31]. Figure 3 shows the fabrication procedure. First, the surface of the Si substrate was activated by O_2_ plasma at 50 W for 5 min (Figure 3(1)). Then, the hydroxy-terminated PS was adjusted to 1 wt % dissolved in microelectronic-grade propylene glycol methyl ether acetate (PGMEA, Showa Denko, Tokyo, Japan). The hydroxyl-terminated PS was spin coated on the Si substrate at 2000 rpm for 30 s. The substrate was then baked at 200 °C for 10 min in an oven. Since both the surface of the Si substrate and the hydroxy-terminated PS have −OH functional groups, H_2_O desorbs, and the Si surface and the hydroxy-terminated PS covalently bond with −O− (Figure 3(2)). After baking, the excess PS that did not form a covalent bond was removed by rinsing with PGMEA. Consequently, the surface of the Si substrate was coated with a thin layer of PS [29]. On this substrate, 1 wt % PS-PDMS dissolved in PGMEA was spin coated at 1500 rpm for 30 s (Figure 3(3)) and baked at 150 °C for 30 min in an oven. Upon being baked, PS-PDMS separated into PS and PDMS. The separated PDMS aggregated to form spheres. The size of the PDMS sphere depends on the ratio of PS and PDMS [29,31]. After the separation process, the surface of this film was covered by several nanometer-thick PDMS film because PDMS has the low surface energy [31], and the PDMS spheres were beneath this thin film (Figure 3(4)). The surface PDMS layer is not likely to be affected by the PS-PDMS ratio because it was very thin. The PDMS thin film was selectively removed by O_2_ plasma at 50 W for 2 s (Figure 3(5)). After removal of the surface PS thin film, the PS between the PDMS spheres was etched with O_2_ plasma at 10 W for 60 s (Figure 3(6)). The PDMS spheres were thus exposed, becoming nanodot array templates with a 20 nm diameter. The PDMS nanodots were arranged at a pitch of 40 nm, as shown in the SEM (scanning electron microscopy) image of Figure 3(6). In Figure 3(6), although slightly difficult, protruding white dots, which correspond to PDMS spheres, can be observed, and the visible dots other than PDMS spheres are Au spatter grains.

### 2.2. Cryogenic Glancing Angle Deposition

**Figure 4:**
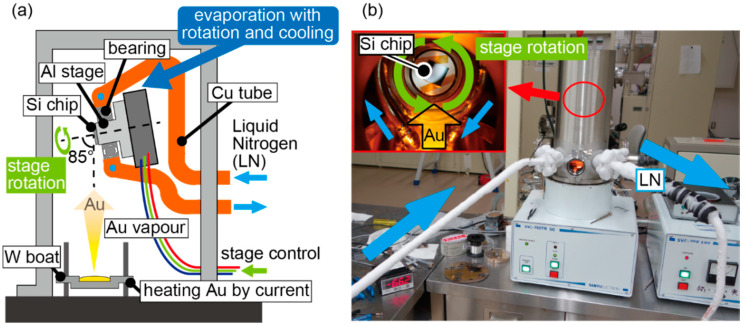
A rotating stage with an aluminium stage was mounted in the vacuum chamber at 85°. A Cu tube, acting as a channel for liquid nitrogen, is bonded to the aluminium stage via a bearing, and rotational deposition can be performed while cooling to −100 °C. (**a**) Schematic of the deposition system. (**b**) Photographs taken during deposition.

**Figure 5:**
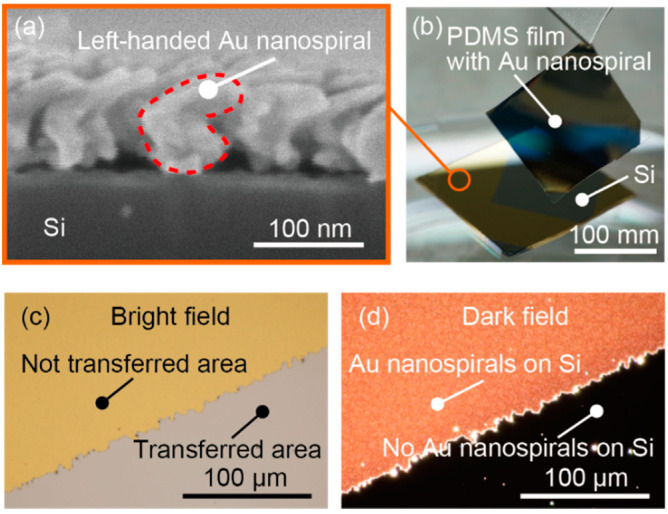
A thin, single-layered circular dichroic filter was fabricated by transferring the Au nanospiral structure fabricated on a Si substrate onto a flexible and transparent PDMS thin film. (**a**) SEM image of a side view of the left-handed Au nanospiral structures with a diameter of 70 nm on a Si substrate. (**b**) Transferred Au nanospiral structures. (**c**) A photo of the substrate after transferred to a PDMS thin film taken with an optical microscope with bright field. (**d**) A photo of the substrate after transferred to a PDMS thin film taken with an optical microscope with dark field.

As shown in Figure 4a, a vacuum deposition system was constructed for cryogenic glancing angle deposition with a stage temperature of −100 °C, stage rotation, and irradiation of Au vapor at 85°. This system was constructed using a commercial thermal evaporation machine (SVC-700TMSG, SANYU Electron, Tokyo, Japan). Most of the system was embedded in a vacuum chamber. The metal vapor is produced by resistive heating with W (Tungsten) boat at the bottom of the system, and the vapor is deposited on the nanodot template Si substrate attached to a rotating stage. A vacuum-compatible stage (VSGSP-60YAW, Siguma-koki, Tokyo, Japan) was used as the rotating stage. An aluminum stage for fixing the nanodot template Si substrate was attached to the rotating stage. The rotating and aluminum stages were mechanically fixed inside the chamber such that the metal vapor reached the Si substrate with an angle of incidence of 85°, as shown in Figure 4a. The top of the aluminum stage was fabricated to be cylindrical, and a ball bearing (MT2-SEB206J1ZZ1C3/0G, NTN, Osaka, Japan) was attached to the cylindrical aluminum stage. Moreover, a copper tube (Cu tube) was looped around the ball bearing and was in thermal contact with the ball bearing via soldering. Then, liquid nitrogen was provided through the Cu tube. In this way, the stages were cooled by liquid nitrogen via the contact between the ball bearing and the aluminum stage, and the handmade vacuum system realized simultaneous rotation and cooling of the Si substrate. The actual evaporation process was performed as shown in Figure 4b. The inset of Figure 4b shows the rotating Si substrate seen from a viewport of the vacuum chamber. Liquid nitrogen was circulated inside the Cu tube, and its flow direction is indicated by the blue arrows. In the fabrication, Au was used as the deposition source material. The stage temperature was approximately −100 °C during evaporation. The chamber pressure was typically 5.0 × 10^−4^ Pa. The stage rotation speed was 0.34 rpm, the deposition time duration was 180 s, and the deposition rate was 0.4 nm/s. During the deposition, Au was solidified and deposited on the PDMS nanodots, and the spiral structure was formed by rotating the stage. After deposition, Au nanospiral structures with a diameter of 70 nm were formed on the Si substrate. An SEM image of a side view of the fabricated nanospirals is shown in Figure 5a. The shape of the nanospiral structures can be adjusted mainly by changing the deposition rate and the stage rotation speed; for example, the diameter increases as the stage rotation speed is slowed down [26]. At this time, nanospiral structures were single turn because the stage was rotated only once. The fabricated film was not a uniform film but contained distinctive structures due to the cryogenic deposition. Focusing on a single structure enclosed by the dotted circle in Figure 5a, the structure is twisted, which can be attributed to the rotating glancing angle evaporation. When the rotation direction of the stage was changed to the opposite direction, the spiral handedness became the opposite handedness. Thus, nanometal spirals were fabricated using cryogenic glancing angle deposition in the handmade vacuum evaporation system.

### 2.3. Transfer of Au Nanospiral Structures onto Polydimethylsiloxane (PDMS) Thin Films

The Au nanospiral structures were then transferred onto isotropic, transparent thin and flexible PDMS thin films to form a film that exhibits transmissive circular dichroism. In the fabrication of the PDMS thin films, the main agent and hardener (Sylgard 184, Dow Corning, Midland, MI, USA) were mixed in a 10:1 ratio and defoamed in a vacuum desiccator for one hour. After defoaming, the PDMS was deposited onto a glass substrate by spin coating at 1000 rpm for 60 s and then heated at 130 °C for 2 h. The PDMS films on glass substrates were cut into 150 mm squares with a scalpel, peeled off, and placed on a Si substrate with fabricated Au nanospiral structures. In general, the bond strength between Au and PDMS is weak, and surface treatment is often performed prior to the transfer of a pattern [33]. However, the Au nanospiral structures could be transferred only by self-adhesion to the surface of the PDMS thin film without such treatment because the PDMS nanodots had weak adhesion to the Si substrate. Applying pressure on the PDMS film to enhance the adhesion between the PDMS film and the Au nanospiral structures was thus not necessary. In this way, a single-layered Au nanospiral-transferred PDMS film was fabricated. The transferred Au nanospirals are shown in Figure 5b. The transferred Au pattern was clearly seen on the surface of the Si substrate, where the Si surface appeared due to the transfer of Au nanospiral structures (Figure 5c). A photo of the substrate after transferred to a PDMS thin film taken with an optical microscope with dark field showed that almost all of the Au nanospiral structures could be transferred to PDMS because a plasmonic red scattered light derived from the Au nanostructures was not observed in the transferred area (Figure 5d). There is possibility that the orientation of the Au nanospiral structures changed during the transfer to the PDMS thin film. This was not an obstacle in the following verification experiments. Because the Au nanospiral structures has chirality even when the orientation changes, the circular dichroism can be observed regardless of the quality of transferal. In a practical use, the orientation will be important. The use of micro transfer printing technique may be promising when transferring the Au nanospiral structure to the PDMS thin film without changing its orientation [34,35].

## 3. Evaluation of Circular Dichroism

**Figure 6:**
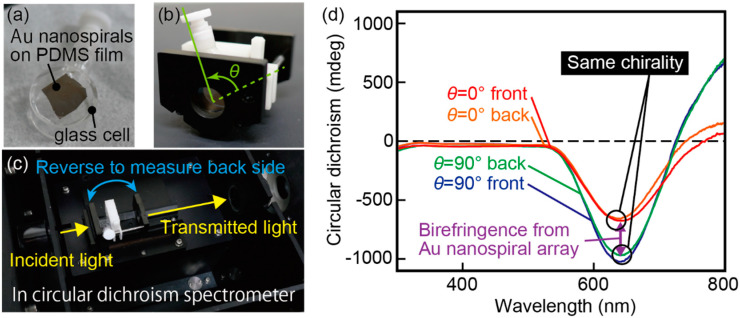
Transmission characteristics when light is incident from the front and back of the device and from the front and back of the device at a 90° tilt. The transmission characteristics of the front and back surfaces at the same angle were almost identical. The circular dichroism measurement error is confirmed by the linearity of the birefringence. (**a**) PDMS thin film with Au nanospiral structures on a glass cell. (**b**) Glass cell holder and tilt angle of the glass cell. (**c**) Setup of the circular dichroism measurement. (**d**) Circular dichroism spectrum of the device which has left-handed Au nanospiral structures for front side and back side illumination with *θ* = 0° or 90°.

The transmission characteristics of the fabricated film were evaluated with a circular dichroism spectrometer (J-720W, JASCO, Tokyo, Japan). The film was mounted on a glass cell such that the light was vertically incident on the film (Figure 6a). The glass cell was then placed on an adjuster to be installed in the spectrometer (Figure 6b). Since the glass cell can be rotated around the light propagation direction, indicated by the yellow lines in Figure 6c, the azimuth rotation angle *θ* of the film can be altered. The angle at which the glass cell contacts the slider of the adjuster was defined as *θ* = 0°, indicated by a dotted line in Figure 6b. The angle *θ* = 0°, or orientation, of the Au nanopiral structure film was arbitrary taken. In the following, *θ* = 90° was defined to be relative to this angle. To measure the circular dichroism without a linear birefringence, we measured the circular dichroism spectra at two azimuth rotation angles, *θ* = 0° and 90°. In addition, circular dichroism spectra were obtained for two light incidence directions, where the light was incident on the film from the front side or from the back side. In this case, the front side was defined as the surface on which the Au nanospirals were formed. The measured spectra are shown in Figure 6d. The measurement wavelength ranged from 300 nm to 800 nm, covering the whole visible wavelength range. The film exhibited strong circular dichroism at approximately 650 nm. The amplitude of the circular dichroism differed depending on the azimuth rotation angle, which can be attributed to the liner birefringence. In contrast, the circular dichroism was almost the same regardless of the incident direction. This result is consistent with the chiral structure’s reciprocal characteristic that the circular dichroism is the same regardless of whether the light is coming from the front or the back [36], so the result is reasonable. The linear birefringence is expected to be due to the Au nanospiral configuration in which the Au nanospiral structures are oriented in the same direction, which can be reduced by averaging the circular dichroism at *θ* = 0° and 90°. In the following measurements, to reduce the linear birefringence effect, the device characteristics were evaluated by averaging the results of four patterns of measurements in which light was incident from the front and back of the film at *θ* = 0° and at *θ* = 90°. Notably, the measurement error may include a component that is independent of the rotation [37], so small errors may still remain in the evaluated results.

**Figure 7:**
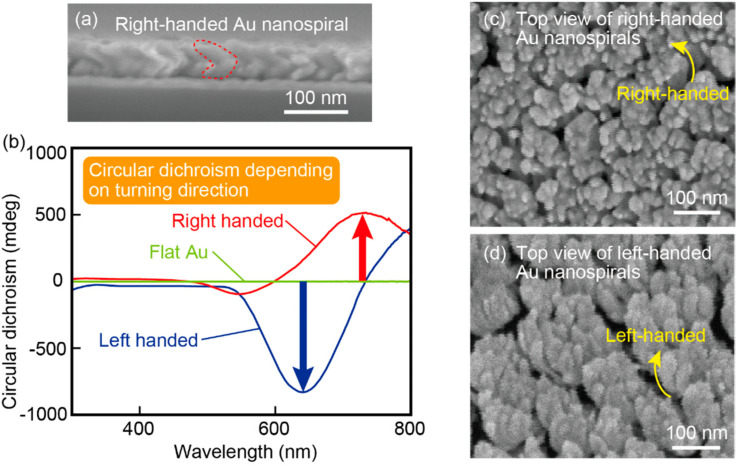
The Au nanospirals with reverse winding directions had opposite polarity circular dichroism to each other, meaning that the filter has chirality. (**a**) SEM image of a side view of the right-handed Au nanospiral structures with a diameter of 50 nm on a Si substrate. (**b**) Circular dichroism spectra of the right- and left-handed Au nanospiral structures and a flat Au film. (**c**) SEM image of a top view of the right-handed Au nanospiral structures. (**d**) SEM image of a top view of the left-handed Au nanospiral structures.

**Figure 8:**
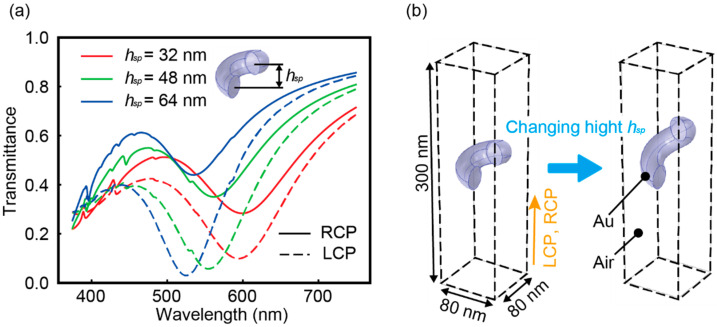
One unit cell of the Au nanospiral structure is modelled with periodic boundary conditions to simulate an infinite 2D array. The right circularly polarized (RCPs) and left circularly polarized (LCPs) were illuminated through the lower port of the unit cell and the transmitted light intensity was acquired at the upper port of the unit cell. (**a**) Transmission spectra of the Au nanospiral structures as a function of height *h_sp_*. (**b**) An overview of the unit cell containing the Au nanospiral structure.

The circular dichroism spectra of the right- and left-handed Au nanospiral structures, which were prepared by changing the rotation direction during cryogenic glancing angle deposition (Figure 5a and Figure 7a), are shown in Figure 7b. Figure 7c is an SEM image of a top view of the right-handed Au nanospiral structures. Figure 7d is an SEM image of a top view of the left-handed Au nanospiral structures. The top view images indicated that the structures are moderately curing. Their curling direction are reversed between the Figure 7c,d, which are corresponding to the handedness of the nanospirals. As a control, the data for a flat Au film with a thickness of 30 nm are also plotted. The flat Au films were fabricated under almost the same conditions as when fabricating the Au nanospiral structures, but the Si substrate did not have a nanodot template and evaporation was performed at room temperature. The flat Au film was almost uniform because a plasmonic red scattered light derived from the Au nanostructures was not observed in the surface with an optical microscope with dark field. No circular dichroism was observed for the flat Au film. Distinctively large circular dichroism was observed for the Au nanospirals. Moreover, the Au nanospiral structures with the opposite handedness exhibited opposite polarity circular dichroism. This result is consistent with the fact that these films had opposite chirality. The amplitude of the circular dichroism for the left-handed Au nanospiral structures showed a negative value peak of −830 mdeg at the 630 nm wavelength. In contrast, the right-handed Au nanospiral structures exhibited a positive value peak of 518 mdeg at 742 nm. Although the peak polarity reversed depending on the chirality of the nanospiral structures, the peak wavelength positions were not the same. This difference can be attributed to the fluctuation of the deposition rate of Au. In the constructed vacuum evaporation system, a deposition rate control system was not installed, so open loop rate control was adopted. Therefore, a slight difference in the deposition rate might have resulted in a spiral height difference between the right- and left-handed spirals, thus producing different resonant frequencies and deviation of the peak wavelengths. The reason why the right-handed Au nanospiral structures, which were smaller in size than the left-handed ones, had a peak on the longer wavelength can be attributed to the height difference of the structure. Figure 8a shows the calculated transmission spectra of the Au nanospiral structures as a function of height of the structure. The simulation was performed using COMSOL Multiphysics 5.5. One unit cell of the Au nanospiral structure is modelled with periodic boundary conditions to simulate an infinite 2D array (Figure 8b). The dielectric constant of Au was defined as a function of the Lorentz-Drude model, and the dielectric constant of air was set to 1 [38]. The size of the unit was set at 80 nm × 80 nm × 300 nm. This corresponds to the Au nanospiral structures spaced 80 nm apart in a 2D plane. The shape of the Au nanospiral structure was as follows. The number of turns was 0.8. The diameter of the rolls, which is the diameter of the nanospiral seen from the infinitely distant point in the longitudinal direction, was 40 nm. The cross section of the nanospiral wire was determined to be circular and the diameter of the wire width was 30 nm. The turning pitch, which is the longitudinal distance for the spiral to take one turn, was 40 nm. The height of the structure was denoted as *h_sp_*, and was 0.8 times of the turning pitch. The RCPs and LCPs were illuminated through the lower port of the unit cell and the transmitted light intensity was acquired at the upper port of the unit cell. The changes in transmission spectra were investigated by varying only the structural height *h_sp_* (Figure 8b). As *h_sp_* increased, the peak wavelength tended to become shorter. This result suggests that the right-handed Au nanospiral structures, which is lower in height than the left-handed ones, had a peak at longer wavelength because of the relatively low height of the structures. As described above, the peak wavelength of the transmission spectrum may change due to changes in the height of the Au nanospiral structures, which is presumably due to difference in the deposition rate. Since this problem can be solved by monitoring the deposition rate, improvement of the evaporation system will solve it. The maximum circular dichroism amplitude obtained in this study, −830 mdeg, is comparable to the results in a previous study [18], where circular dichroism of ~2 deg was observed for chiral meta-fluids with an optical path length of 1 cm. The thickness of the active layer with respect to the circular dichroism of the proposed spiral-transferred PDMS film was only approximately 100 nm, so the Au nanospirals transferred onto the PDMS film were confirmed to exhibit significantly large circular dichroism per medium thickness at visible wavelengths.

**Figure 9:**
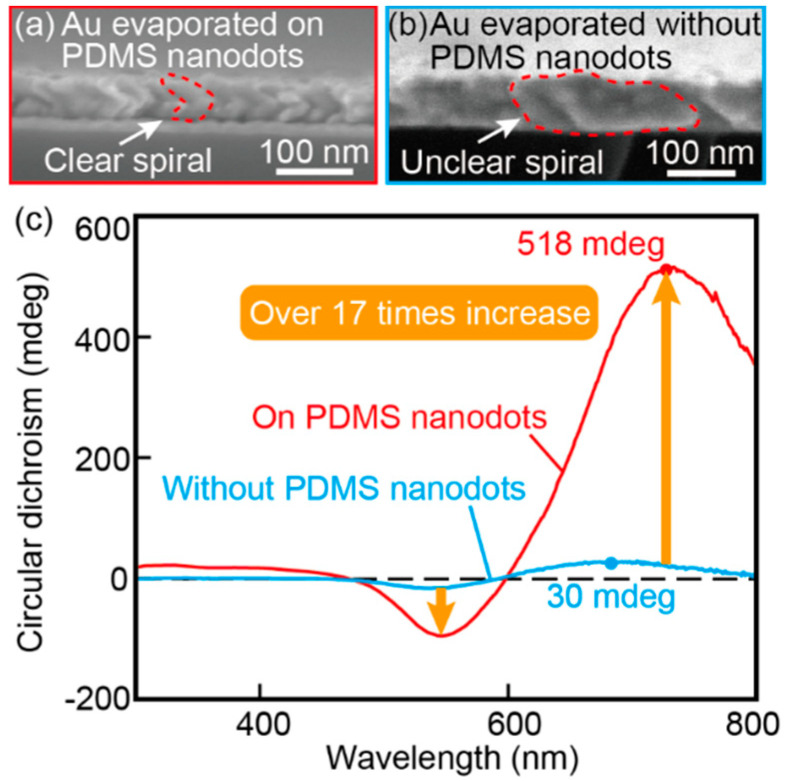
SEM images showing that the PDMS periodic nanodot template is important for the formation of fine Au nanospiral structures. When focusing on the circular dichroism, the condition with a template produced a peak that was 17 times larger than that for the condition without a template. (**a**) SEM image of a side view of the right-handed Au nanospiral structures evaporated on a PDMS nanodot template. (**b**) SEM image of a side view of the right-handed Au nanospiral structures evaporated without a template. (**c**) Circular dichroism spectra of the two cases with and without the template.

Furthermore, Au nanospiral structures fabricated with and without a dot template were transferred onto PDMS thin films, and their transmission characteristics were measured to investigate the role of the nanodots in generating circular dichroism (Figure 9). Figure 9a,b shows SEM images of a side view of Au nanospiral structures before transferred to a PDMS film. Figure 9a shows Au nanospiral structure with the nanodot template and Figure 9b shows Au nanospiral structure without the nanodot template. From Figure 9b, we can see that under the condition without the nanodot template, many structures are connected to each other and do not form clear nanospiral structures. In the absence of the nanodot template on substrate, Au tended to form a continuous film because Au particles which formed on the substrate as the vapor solidified with very small diameter at random locations were less likely to shadow each other. In both cases, stronger circular dichroism than those natural materials was observed [2,3]. However, the film fabricated with the nanodot template showed over 17 times stronger circular dichroism than the non-template film. These results indicate that the nanodot templates assisted the formation of fine Au nanospiral structures, providing strong circular dichroism.

## 4. Conclusions

We proposed a thin, single-layered circular dichroic film with Au nanospiral structures on a PDMS thin film that has strong circular dichroism at visible wavelengths. Au nanospiral structures with a diameter of 70 nm were fabricated by cryogenic glancing angle deposition on a Si substrate with a nanodot array template patterned with the block copolymer PS-PDMS. The Au nanospiral structures were transferred onto a transparent and flexible PDMS thin film to fabricate a thin, single-layered circular dichroic film. The film had a very large peak amplitude of circular dichroism of −830 mdeg at 630 nm. The results showed that the strong circular dichroism of Au nanospiral structures in the visible light region can be transferred to thin circular dichroic films. This study showed that the nanometal spirals can be applied for optical filter use. The nanospiral structures in this report were not clear and the structures are distorted ones. To obtain clearer spiral shapes, finer patterning of PS-PDMS is necessary. This will be possible by tuning the nanodot formation process. Electron beam lithography will provide highly aligned nanodot templates. Moreover, precise control of the thermal evaporation rate is indispensable for fabricating finer spirals. In addition, if the chiral structure is arrayed in the form of C_4_ rotation symmetry, then birefringence with respect to linear polarization can be eliminated in principle [39]. The combination of nanospiral formation and microfabrication technology that aligns spirals in such symmetrical manners will provide a new way to construct practical metamaterial-based optical components.
